# Twenty-nine years of continuous monthly capture-mark-recapture data of multimammate mice (*Mastomys natalensis*) in Morogoro, Tanzania

**DOI:** 10.1038/s41597-023-02700-3

**Published:** 2023-11-11

**Authors:** Herwig Leirs, Lucinda Kirkpatrick, Vincent Sluydts, Christopher Sabuni, Benny Borremans, Abdul Katakweba, Apia Massawe, Rhodes Makundi, Loth Mulungu, Robert Machang’u, Joachim Mariën

**Affiliations:** 1 Evolutionary Ecology Group, Department of Biology, University of Antwerp, Antwerp, Belgium; 2 Institute of Pest Management, Sokoine University of Agriculture, Morogoro, Tanzania; 3 Department of Ecology and Evolutionary Biology, University of California, Los Angeles, CA USA; 4 Department of Biology, Royal Museum for Central Africa, Tervuren, Belgium

**Keywords:** Population dynamics, Tropical ecology

## Abstract

The multimammate mice (*Mastomys natalensis*) is the most-studied rodent species in sub-Saharan Africa, where it is an important pest species in agriculture and carrier of zoonotic diseases (e.g. Lassa virus). Here, we provide a unique dataset that consists of twenty-nine years of continuous monthly capture-mark-recapture entries on one 3 ha mosaic field (MOSA) in Morogoro, Tanzania. It is one of the most accurate and long-running capture-recapture time series on a small mammal species worldwide and unique to Africa. The database can be used by ecologists to test hypotheses on the population dynamics of small mammals (e.g. to test the effect of climate change), or to validate new algorithms on real long-term field data (e.g. new survival analyses techniques). It is also useful for both scientists and decision-makers who want to optimize rodent control strategies and predict outbreaks of multimammate mice.

## Background

Nearly one century after Elton’s seminal work on periodic cycles and outbreaks of animal populations^1^, ecologists remain intrigued by their causes and consequences^2–4^. The phenomena are particularly well-studied for rodent populations, as they can be collected in large numbers, are easily manipulated in experiments, and may cause important economic and public health problems^5,6^. Long-term data series are a particularly valuable tool when understanding the processes underpinning how rodent population fluctuations develop, and can therefore be essential for predicting population outbreaks. While such data can be found from several locations in Eurasia and North America^5,7^, long-term data are rare from more Southern latitudes^5,8,9^ and especially so in sub-Saharan Africa, where limited resources and logistical challenges impede the continued collection of long-term data even more^10^.

One of the most-studied rodent species in sub-Saharan Africa is the multimammate mouse, *Mastomys natalensis* (family *Muridae*, subfamily *Murinae*). The animal owes its name to the two rows of 12 mammae that females possess, corresponding to the large litter size at birth (up to 24 pups, but 11 on average)^11^. Their natural environment is savannah and grassland, but *M. natalensis* also flourishes in agricultural fields and human dwellings^12^. In agriculture, *M. natalensis* is considered the most important rodent pest species in Africa, as outbreaks can cause crop losses up to 80% at both household and regional levels^13,14^. These rodents are also hosts for several pathogens causing human diseases, including Lassa fever, bubonic plague and leptospirosis^15–18^. Outbreaks of zoonotic diseases can be predicted based on the population dynamics of this rodent reservoir^19^. For example, outbreaks of Lassa fever occur mainly at the end of the dry season in West Africa (January-April), when most *M. natalensis* can be found in the houses of rural villages^20–22^.

Over the past 40 years, the ecology of *M. natalensis* in Morogoro (Tanzania) has been intensely studied because of its importance to agriculture in the region^11,14,23–25^. In this area, *M. natalensis* population dynamics are highly dependent on a bimodal rainfall pattern with long (March-May) and short (November-December) rains^40^. The variation in rainfall results in strong density fluctuations between seasons, ranging from 20–300 individuals per hectare^11,24^. In Tanzania, the reproduction of *M. natalensis* starts at the end of the long rainy season when grass sprouts and continues until November^25,26^. Thereafter, the population declines due to lack of food and intra-specific competition, to arrive at its lowest point around May^25^. Sporadic population outbreaks may happen after periods of heavy rainfall early in the new year, as this stimulates the growth and fecundity of new recruits. This results in mice from two generations being able to reproduce in the same season, leading to outbreaks of up to 1000 individuals per hectare in some years^25,27^. Other interesting aspects of this species include its promiscuous behaviour and lack of territoriality which is rarely observed in other rodent species^28–30^. Their home range size is generally considered to be small, although the observed size largely depends on the methodology used (e.g. ± 650 m^2^ by capture-mark-recapture and ±1200 m^2^ by radio-tracking methods)^28,31^. Finally, we want to make the reader aware that the population dynamics of *M. natalensis* can differ in other regions of Africa^20^.

In the past, demographic information extracted from the long-term time series has been used to build ecologically-based rodent control models to predict outbreaks of *M. natalensis* and optimize pest management strategies^22,32,33^. Here, twenty-nine years of continuous monthly capture-mark-recapture data of *M. natalensis* in Morogoro is presented; at the time of publication, monthly captures are still ongoing. This is one of the most accurate and long-running capture-recapture time series on a small mammal species worldwide which can be used to estimate various demographic parameters. We believe this dataset will allow researchers to estimate demographic parameters upon which population models can be built (e.g. to develop more efficient management strategies for pest species), and to test ecological hypotheses on the causes and consequences of rodent population dynamics.

## Methods

### Ethics statement

All the procedures followed the Animal Ethics guidelines of the Research Policy of Sokoine University of Agriculture as stipulated in the ‘Code of Conduct for Research Ethics’ (Revised version of 2012) and the guidelines in Sikes and Gannon^34^ since ethical permits were required. The used protocol was approved by the University of Antwerp Ethical Committee for Animal Experimentation in subsequent projects (2014–44, 2015–69, 2021–04) the ethical committee of the Sokoine University of Agriculture (A.M. Lupindu, chairperson ethics committee) and adhered to the EEC Council Directive 2010/63/EU.

### Context of the project

H. Leirs and colleagues started a long-term capture-mark-recapture study in March 1994 on one 3 ha grid to study the population dynamics of *M. natalensis*, which, after 29 years of monthly capture-recapture, is still ongoing. The data contains individual trapping histories of rodents, their sex, species, body weight, trapping coordinates and sexual condition. East Africa, and in particular Tanzania, has a long history of rodent problems^11,24^. Despite substantial inputs of time and resources, efforts to control rodent populations and reduce damage were rarely effective, and the Tanzanian government realized that more knowledge about the biology of the pest species was needed. Foreign expertise and financial inputs were sought and in 1980 the Denmark-Tanzania Rodent Control Project started, linked to the Ministry of Agriculture. In March 1986, a second initiative, the “Tanzanian-Belgium Joint Rodent Research Project on the role of Rodents as Carriers of Disease and Destroyers of Agricultural Crops” started. The Tanzanian partner was Sokoine University of Agriculture (SUA) where the project was attached to the Faculty of Veterinary Medicine. In Belgium, the Laboratory of General Zoology (currently the Evolutionary Ecology group) at the University of Antwerp was entrusted with the execution of the project. Both of those early projects carried out relatively short (<2.5 years) capture-recapture studies. The study under the Tanzania-Belgium project (from November 1986 until February 1989) was a precursor for the long-term study we are reporting here. Indeed, the collaboration between the Tanzanian and Belgian rodent researchers persisted through consecutive projects paid for by various funding sources and contributed to what nowadays has become the SUA Institute of Pest Management.

### Description of the location and the study area

**Fig. 1:**
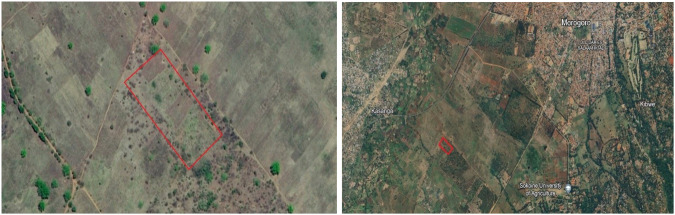
Satellite pictures of the MOSA field. The red rectangles represent the boundaries of the field (© Google Earth).

**Fig. 2:**
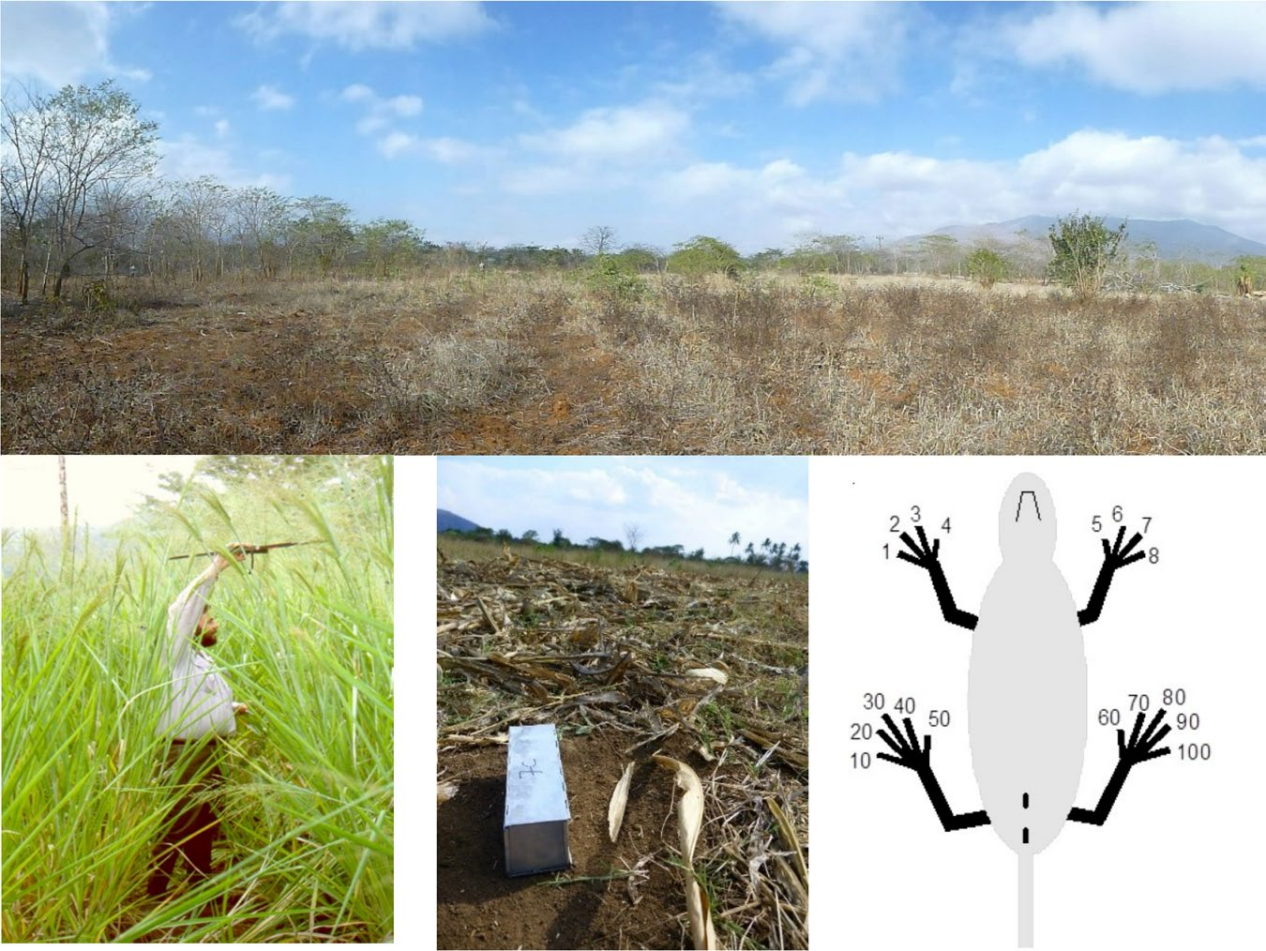
Top: a view on the MOSA field towards the end of the dry season in September 2013. Bottom left: H. Leirs trapping *M. natalensis* after the end of the wet season in July 1996. Bottom middle: a Sherman live trap in the field. Bottom right: schematic view of the labelled toes for toe-clipping.

The study area is located on the campus of SUA in Morogoro, Tanzania (Fig. 1). The campus is located on the northern edge of the Uluguru mountains at an elevation of approx. 650 m. The original vegetation on the campus has largely been replaced by agricultural or fallow land. The university buildings are located on the slopes of the mountains but our field is located in the plains, just outside the main university entrance gate. As would be expected from a university focussing on agricultural research, there is a very large campus with a lot of land used for the experimental growing of crops or raising cattle. However, not all fields are cultivated, as they are often left fallow after some years of maize growing, which is the primary crop in the area. The main grass species on this fallow land are*: Panicum hanningtonii* Stapf, *Rottboellia conchinchinensis* (Lour.) Clayton, *Pennisetum polystachyon* (L.) Schult. and *Cymbopgon spp*. in varying relative importance. Trees are sparsely present, most often Kapok (*Ceiba pentandra*) or *Acacia* spp. Bird observations and pellet data clearly showed that black-shouldered kites and barn owls were the main avian predators of *M. natalensis* in the area^35^. These predators were not found to significantly affect *M. natalensis* densities in Morogoro^36,37^. Our grid is located on a mosaic field (6°51′S and 37°38′E), which is managed to be partly fallow and partly planted with maize, hence the name of our study field ‘MOSA’ (Fig. 2 top). Although the overall proportions have changed slightly over the years, around one-third of this field is annually planted with maize and two-thirds are left to be fallow land. Bushes and trees are cleared now and then to maintain the fallow vegetation. A couple of times since the start of the study, the area was burned by wildfire escaped from nearby farmer fields. While the field itself remained largely the same over time (see Zenodo ‘pictures MOSA.pdf’^38^), the surrounding fields changed due to the increased urbanisation of Morogoro city. For example, the closest buildings to the field are currently approx. 2 km away from the field (>5 km when the experiment started). We assume that this resulted in reduced water levels in the surrounding fields as more water is used by the city, resulting in potential land-use change. Similarly, increased urbanisation might also have resulted in more domestic rodent predators, such as cats and dogs (although they are not frequently observed around the field).

### Trapping design

The study setup follows a robust design with primary capture sessions each month containing three consecutive nights (secondary trapping sessions) across one 3 ha grid. Rodents are captured with Sherman LFA Live traps (from H.B. Sherman Traps, Inc., Tallahassee, FL, USA), which are aluminium rectangular boxes (7.5x9x23cm) with a door that closes when the animal enters the trap and steps on a baited triggering platform (Fig. 2 bottom middle). The animal is thus trapped alive and without harm. The grid consists of 30 parallel lines, 10 m apart, with every 10 m a trapping station, resulting in 300 trapping stations. Each trapping station is identified by coordinates A to J, 1 to 30. Traps are placed late afternoon on the first day of a new primary capture session, baited with a mixture of peanut butter and maize flour and checked each morning before 9AM.

### Marking technique

Since the start of the capture-recapture studies in Morogoro, all captured animals are individually marked by “toe-clipping” (rather, clipping of terminal phalanges)^34^, and this method so far has been maintained for consistency (Fig. 2 bottom right). The toes are numbered 1–4 and 5–8 on the right and left front feet respectively and 10–50 and 60–100 on the right and left hind feet respectively, always counted from left to right when the animal is held on its back (see figure). The marked toes are always read in the same sequence and are typed without any spaces or symbols between the toes in the data file, *e.g*. 26106090. If an animal is very peculiar, *e.g*. once an animal was caught that had only three legs, then the toe clipping code may be some words, *e.g*. “no left front leg”. Although newer techniques exist, toe-clipping is best suited for fieldwork in the tropics since it is cheap, easy and independent of supplying materials from abroad or electricity. The studied *Mastomys* mice are of sufficiently small size to allow the clipping without trauma. We also found that toe-clipping itself has no detectable effect on the survival of the animals^38^.

## Data Records

**Fig. 3:**
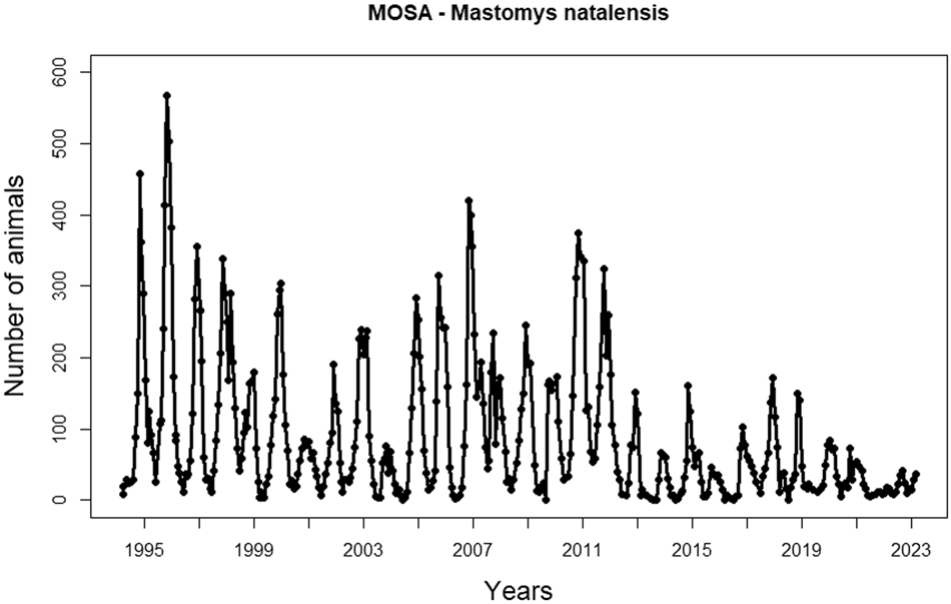
Number of captured *Mastomys natalensis* individuals from the monthly capture-mark-recapture on a 3 ha field in Morogoro (Tanzania). Dots represent the number of unique captured animals per primary trapping session.

All data from March 1994 until February 2023 can be downloaded from Zenodo^38^ from the file “**mosa_Feb2023_Flags.csv**” (https://zenodo.org/record/10049811). For each trapped animal, nine individual characteristics were recorded and entered into a specifically designed MS Excel-based program ‘CMRINPUT’ (Zenodo files: “CMRINPUT empty.xlsm” and “CMRINPUT Manual.pdf”). The data are stored as “capture records”, each capture record being the information collected during a single capture occasion of a given individual. From March 1994 until February 2023, the total dataset consisted of 49216 captures of 21927 unique individuals. The majority of rodents captured in the field are *M. natalensis* (*n* = 45433 of 19850 unique individuals) (Fig. 3). Other captured species were C*rocidura hirta* (n = 56 captures), *Lemniscomys rosalia* (n = 1441 captures), *Mus minutoides* (n = 44 captures), *Dasymys sua* (n = 1 capture) and *Gerbilliscus vicinus* (n = 2033 captures).

Here, we provide a summary of all the individual characteristics that are entered per captured animal in the database:

- ***DATE*** The date of the capture (the morning when the traps were checked).
- ***GRID*** Name of the field. In our case, this is always MOSA.
- ***SPECIES*** Any of the accepted species name abbreviations defined in the worksheet “setup”. These are the rodents that we trap in that field.

MN *Mastomys natalensis*

TA *Gerbilliscus sp.* (previously called *Tatera sp.*) – [in this study site *G.vicinus*]

LR *Lemniscomys rosalia*

CH *Crocidura hirta*

MM *Mus minutoides*

RR *Rattus rattus*

XX other species, name added to the COMMENT field [e.g. in this study 1 *Dasymys sua*]

*X:* X-coordinate of the trap 1–30.

*Y:* Y-coordinate of the trap A-J.

*WEIGHT:* Body weight in g measured via pesola or a digital balance (1 g).

*TOE-ID:* The toe clipping code or other unique identification for the captured individual. As we only clipped a limited number of toes, a new series of codes was started a few times, but always with several years in between series so that mixing up individuals was not possible; a prefix was added before each toe-code to indicate the series (e.g. 5/1520, indicates that this is a fifth individual with the toe-code 1520 in our dataset).

*SEXCON:* A code for the reproductive condition of the animal. This a is a two-letter code for males, a three-letter code for females. The codes are formed as follows:

males:    1st letter: Position of the testes **A** (abdominal) or **S** (scrotal)

2nd letter: epididymal gubernacula **N** (not visible) or **V** (externally visible)

females: 1st letter: Vagina **C** (closed) or **P** (perforated)

2nd letter: nipples **S** (small) or **L** (lactating)

3rd letter: pregnancy status **N** (not visibly pregnant) or **Y** (visibly pregnant)

Interpretation of sexual condition: Males are considered to be sexually active when, at external examination, the testes are scrotal and the gubernacula visible. Males with these characteristics have mature spermatozoa in the spermatic tubules. In females, the external characteristics are less obvious and the correct recording depends greatly on the observer’s experience. Since several people participated in the data collection in the CMR grid, the data should be treated with caution. Moreover, even an experienced observer cannot detect early pregnancy by palpating the abdomen. Females are considered to be sexually active if the vagina was perforated, the nipples swollen or the female visibly pregnant.

We included the following files in the repository^38^.

- **mosa_Feb2023_flags**: the raw CMR data csv file since 1994, including flags for errors that were corrected from the field list.
- **C_primary_secondary_capture_3consecutive_o_1_10000b.csv** and **C_primary_secondary_capture_3consecutive_o_10000**–**20000b.csv**: these two csv files provide the encounter matrix that is used in the Rmarkdown file 3. We had to split one original file in two because Zenodo only allows to upload files until 25MB.
- **C_primary_secondary_time_variables_3consecutive_m2.csv**: A csv file with all the time variables per capture session that is used in the Rmarkdown file 3
- Four different **RMarkdown files** that provide an introduction to the system (1), how we processed the data (2), a density and survival analyses (3) and serological information (antibodies against Morogoro arenavirus) of the rodents^19^.
- **CMRINPUT excel and manual**: CMRINPUT is an MS Excel-based program to facilitate the computer storage of CMR-Data. The data are stored as “capture records”.
- **A PowerPoint presentation (pictures MOSA.pdf):** with pictures of the mosa field over time.

## Technical Validation

The CMRINPUT program provides easy input screens and has several routines to verify the validity of the entered data and intercept erroneous input, based on the characteristics of the study area and its species composition. To ensure the validity of the input data, the files are protected against changes. The structure and format of the worksheets and the program itself are protected, and data can only be entered using the program buttons (see below) to reduce the risk of mistakes occurring during data input. There is no facility for performing data analysis of the recorded data within the program, to carry out analysis the data must therefore be copied to another Excel file and manipulated from there. In this way, the quality of input data are maintained.

Despite these checks, errors can still occur. We instigated routines (based in R) to identify and correct these errors, while maintaining the original data. An example of an error we might detect is a toe clipping code conflict, for example when two or more animals simultaneously had the same toe clipping code. This can be detected when such animals are trapped on the same day, or when we noticed from the capture history that a certain toe clipping code often changes sex, species, weight or home range. If detected, we would adapt the toe code as follows:

- First, we inspect the raw data (paper documents) on which the field data are collected. We double-check the toe clipping code but also sex, trap coordinates, etc. It may be that we previously made an error in identifying the sex (challenging to determine in young animals) or the species of the animal, for example, which can be updated by adding a new capture record with the correct data and a note in the *Remarks* field explicitly confirming the correct observation. We never change the previous capture record, unless we have proof of our mistake there (*e.g*. when paper notes indicated data entry errors).
- If we are sure that there are two different animals with the same toe clipping code, there are two possible solutions. If the two animals could be easily and permanently recognised (because they are of different sexes or belong to different species), we kept the toe clipping code for both animals. For one of them, however, we added a letter “B” to the toe clipping code *e.g*. 4630100 becomes 4630100 B). In all other cases, we clipped one or more additional toes of one of the animals so that the animal was recognised in the future by another toe clipping code. We then changed the TOE-ID to the one that we actually saw on the animal.
- After the data input in the field, all data were checked a final time for obvious mistakes using an R-script (MOSA website). Potential errors were corrected as follows: If captures with the same toe-code exceed two years, we added the letter “B” to the toe-clipping code after year two. We did not find that *M. natalensis* is older than 9–12 months in the field (average life expectancy is 3 months), so we assumed that this must be two separate individuals^11^.
- If captures with the same toe-code have a different sex, we correct the sex if the mistake is obvious. For example, if the previous four times the toe code indicates the individual captured was AN (male, reproductively inactive) and once CSN (female, reproductively inactive), we change the CSN to AN. In other cases where this is not possible to determine, the sex is set to NA in the dataset
- If the body weight at a consecutive capture was more than 50% different from the body weight at a previous capture, we considered this to be a mistake and wrote NA in the data file.
- We included a column with flags if errors were found and corrected from the initial field list.

*CON*: sexual condition

*DAT* date

*WE*: body weight

*SX*: sex

*SP*: species

## Usage Notes

To the best of our knowledge, this database constitutes the longest time series of continuous monthly capture-recapture data of rodents in Africa. The database can be used by ecologists to test hypotheses on the ecology of small mammals related to population dynamics (e.g. to test the effect of climate conditions or habitat use)^24,25,39,40^, territoriality^41^, social networks^42,43^, infectious disease transmission^19,44,45^ or to validate new algorithms on real long-term field data (e.g. new survival analyses techniques)^46^. It is also useful for both scientists and decision-makers who want to optimize rodent control strategies and predict outbreaks of *M. natalensis*^47^. We highlight that our dataset comes from one field only, which should be taken into account by users who wish to study long-term trends (e.g. it could be more useful to combine this dataset with other long-term studies when investigating climate change effects).

The dataset will be updated yearly and the latest version can be found on the website of the University of Antwerp of Zenodo^38^. We also provide R-Markdown files to clean and further analyse the data, which can be downloaded via the same website. This R-code will allow to:

- Make an encounter matrix (e.g. 101001) which is often used for survival analyses.
- Create a data file with all the relevant time variables (date of trapping session, time between primary trapping sessions, time since the start of the study) and number of the primary or secondary trapping sessions.
- Estimate densities (number of rodents per 3 ha) based on Minimum Number Alive, Schnabel, closed and open CMR models.
- Perform a basic survival analysis using RMark^48^.

## Data Availability

R-code to analyse the data is made available on the website of the University of Antwerp and via the Zenodo repository^38^ (10.5281/zenodo.10049811) under the creative Commons license option (CC-BY). We added the open license to our dataset page (CC-BY). This license enables reusers to distribute, remix, adapt, and build upon the material in any medium or format, so long as attribution is given to the creator. We encourage other researchers who use our dataset to share their code with us. In that way, we can group all analyses that are performed on this MOSA dataset together.
